# Supplementary material for: Genomic characterization of group B Streptococcus from Argentina: insights into prophage diversity, virulence factors and antibiotic resistance genes
Source: Microb Genom. 2025 Apr 23;11(4):001399. doi: 10.1099/mgen.0.001399 (PMC12046356; doi:10.1099/mgen.0.001399)
Supplement: Supplementary Material 1. [file mgen-11-01399-s001.pdf]

Supplementary Results

Detailed presence of VDs in the context of GBS epidemiology

We analysed the presence of diverse known GBS VDs including those involved in immune response evasion (IRE-VD), tissue damage (TD-VD) and adherence to host tissues and invasion (ATI-VD) (Figure 3, Supplementary Figure 6).

IRE-VD

The gene *hyl*B, coding for a hyaluronidase involved in GBS survival inside the macrophage and inhibition of proinflammatory cytokines (Wang et al. 2014), was found in the majority of isolates (89%) except for CC19/III, where it was found in only 1/48 (2%). Accordingly, in Argentinian isolates, *hyl*B had significantly (p<0.001) lower prevalence in niGBS (4%) than in the other collections (more than 84%). The lack of expression of *hyl*B in GBS CC19/III and/or niGBS has already been explained by its interruption by the insertion sequence IS1548 (Rolland et al. 1999; Granlund, Michel, and Norgren 2001; Yildirim, Fink, and Lämmler 2002; Al Safadi et al. 2010; Emaneini et al. 2016; Jamrozy et al. 2023).

The gene *scp*B, which encodes the streptococcal complement inhibitor C5a peptidase (Lynskey et al. 2017), was found in more than 95% of isolates, with the exception of CC1/V where it was present in a significantly (p<0.001) lower proportion (40%), as previously described (Tsai et al. 2022). No significant difference was found in its prevalence among the different GBS collections.

TD-VD

All isolates, except for a CC1/V niGBS, carried the *cfa*/*cfb* gene, which encodes the pore-forming toxin CAMP factor (Schneewind, Friedrich, and Lütticken 1988).

The *cyl* operon genes, that intervene in the beta hemolytic and cytolytic activity of GBS (Pritzlaff et al. 2001), were present in all of the isolates, with the exception of *cyl*A, which was absent in 4 CC19/III isolates from different collections and *acp*C, absent in a CC19/II niGBS isolate.

ATI-VD

The genes coding for the surface proteins of the Alp family, involved in GBS adherence to the host tissues, were distributed distinctively among the different lineages: *alp*1 was predominantly found in CC23/Ia; *alp*2/3 in CC1/V and CC23/II; alpha in CC12/Ib and CC452/Ia, *rib* in GBS from CC17/III, CC19/III and CC19/II. These results are in accordance with previous reports (Jamrozy et al. 2023). Among the Argentinian collections, *rib* prevalence was significantly (p<0.01) lower in niGBS (12%, against over 22% in the other collections). Although the presence of *rib* has been described as not variable according to the status or the age of the patients (Manning et al. 2006; Eskandarian et al. 2015), lower frequency in niGBS could be due to the lower prevalence of serotype III isolates in that collection.

The adhesin coding gene *srr*2 was prevalent in CC17/III isolates, while *ssr*1 was the prevalent one in the rest of the lineages, in accordance with previous reports (Seo et al. 2013).

The gene *hvg*A, which encodes the hypervirulent GBS adhesin (HvgA) associated with CC17/III GBS and their higher invasiveness in the central nervous system (Tazi et al. 2010;

2019), was found almost exclusively in this lineage. No significant difference was found among Argentinian GBS collections, in accordance with the homogeneous distribution of CC17/III strains in each collection.

The genes *fbs*A and *fbs*B, encoding fibrinogen-binding proteins that promote colonisation and invasion (Rosenau et al. 2007), were absent in more than 96% of CC19 and CC12 isolates. The *fbs*A gene was found mainly in CC1/V isolates, while *fbs*B was found almost exclusively in CC17/III, CC23/Ia, CC23/II, and CC452/Ia isolates. Reports on the prevalence of these genes in the various GBS lineages are variable, but *fbs*B has been described to have an important role in the capacity of the hypervirulent CC17 lineage to invade the central nervous system in infants (Rosenau et al. 2007; Safadi et al. 2011; Udo, Boswihi, and Al-Sweih 2013; Bobadilla et al. 2021).

The *lmb* gene encodes the laminin-binding protein, which is involved in the adhesion of GBS to the extracellular matrix, promoting colonisation and invasion (Bobadilla et al. 2021). This gene was found in 98% of the isolates (absent in 7/7 CC103/Ia, 1/4 CC26/V and 1/1 CC327/Ia), as expected (Al Safadi et al. 2010).

Genes involved in the synthesis of GBS pilus were distributed in different pilus islands (PI): *srt*C1, *srt*C2, *gbs*0628, *gbs*0632 and *gbs*_rs03570 were encoded in PI-1 and PI-2b, while *pil*A, *pil*B, *pil*C, *srt*C3 and *srt*C4 in PI-2a. PI distribution among isolates varied according to lineage, in accordance with previous reports (Madzivhandila 2013; Martins et al. 2013): more than 93% of CC17/III isolates presented PI-1a and PI-2b; more than 99% of CC12/Ib strains carried PI-2a, accompanied by PI-1a in half of the cases and by PI-1b in the other half; in CC23/Ia and CC452/Ia GBS only PI-2a was found, while all CC23/II carried both PI-2a and PI-1a and 3/3 CC452/IV presented PI-2b; in more than 89% of isolates of CC1/V, CC19/III and CC19/II both PI-1a and PI-2a were found, while all CC1/VI isolates carried PI-1b. When analysing Argentinian GBS by collection, *srt*C3 was found in a significantly (p<0.01) higher proportion in isolates from invasive infections (92%) compared to those from urinary infections or colonisation (56% and 53%, respectively). The opposite was observed in the case of PI-2b, with 15% prevalence in invasive GBS versus 32% and 28% in urinary or colonisation isolates, respectively.

Discrepancies in ARDs presence and phenotypic antibiotic resistance in Argentinian GBS

ARDs to macrolide, lincosamide and streptogramin B (MLS) class

Discrepancies were found in 11/365 genomes (Supplementary Table S5).

Supplementary Table S5. Discrepancies in Argentinian GBS between the content of MLS ARDs and their phenotypic resistance profile

Phenotypic resistance profile	Isolates (n)	MIC ERY (µg/ml)	MIC CLI (µg/ml)	ARD	% identity ARD detection	% coverage ARD detection
	1	0.25	0.03	*erm* (A)	99.86	100
	1	0.12	0.25	*lsa* (C)	94.52	100
ERY^S-CLI^S	2	0.06/0.12	0.06/0.12	*erm* (B)	99.48/99.61	100
	1	0.03	0.03	*erm* (B), *mef* (A), *msr* (D)	99.48, 100, 100	100
	1	0.06	0.03	*mef* (A), *msr* (D)	100	100
ERY^R-CLI^S	1	2	0.06	Not detected	-	-
ERY^S-CLI^R	2	0.06/0.12	8/2	Not detected	-	-
ERY^R-CLI^R	2	>8	>8	Not detected	-	-

MIC: Minimum inhibitory concentration; ERY: erythromycin; CLI: clindamycin; ARD: antibiotic resistance determinant.

Six out of the eleven discrepant isolates were phenotypically susceptible to erythromycin (ERY) and clindamycin (CLI) but presented ARDs to either ERY, CLI or both.

-In the case of the 2/6 isolates with *erm*A or *lsa*C, it is observed that the MIC value for ERY or CLI, respectively, is a dilution lower than the cut-off value for intermediate susceptibility (0.50 µg/ml) for said antibiotics. For both cases, a disk diffusion test had also been performed, resulting in values at the cut-off point between susceptibility and intermediate susceptibility. This leads to think that these isolates may have an intermediate susceptibility to ERY or CLI, product of the presence of *erm*A and *lsa*C, respectively, and that the assignment of a susceptible phenotype was due to the margin of error characteristic of the tests used for said determination.

-In the remaining 4/6 isolates, it is observed that the MIC values are several dilutions lower than the cut-off value, so the discrepancy between the phenotype and genotype could not be explained by the intrinsic errors of the methodology. In the 4 cases, phenotypic resistance to ERY would have been expected, especially in the 2 isolates where *mef*A and *msr*D were detected with 100% identity and coverage compared to the reference.

In the 5/11 GBS with no ARDs but phenotypic resistance to one or both antibiotics, no ARD was detected with lower identity and coverage thresholds (30%), indicating that the resistance might be mediated by novel genetic variants.

ARDs to aminoglycoside class

Discrepancies were found in 2/135 genomes.

One of the discrepant isolates was phenotypically resistant to high levels of streptomycin (STR) but no ADR was found, suggesting that the resistance might be due to a novel ARD.

The second GBS with discrepancy was an isolate phenotypically resistant to high levels of gentamicin (GEN) but susceptible to STR that carried the genes *aac*(6')-*aph*(2"), *ant*(6)-Ia and *aph*(3')-III. STR resistance would have been expected due to the presence of *ant*(6)-Ia. Analysing the ARD search results, it was noted that *ant*(6)-Ia was detected with 100% identity and 88% coverage in one contig (over the threshold for ARD detection) and 100% identity and 12% coverage in the same contig but upstream, so the contig was analysed and it was found that the *ant*(6)-Ia gene was interrupted by the *aac*(6')-*aph*(2") gene. This interruption could explain the lack of phenotypic resistance to STR in this strain.

ARDs to quinolone class

Discrepancies were found in 10/365 genomes (Supplementary Table S6).

Supplementary Table S6. Discrepancies in Argentinian GBS between the phenotypic resistance profile to quinolones and the detection of point mutations in the *gyr*A and *par*C genes *in vitro* and *in silico*

Phenotipic resistance profile	Isolates (n)	Mutation detected *in silico*		Mutation detected by PCR	
		gyr A	*par* C	*gyr* A	*par* C
$(LEV-NOR)^S$	1	-	S79F	Not determined	
	1	-	S80P		
	1	S81L	-		
	3	S81L	S79F		
$(LEV-NOR)^R$	1	-	-	Not determined	
$(LEV-NOR-CIP-PF-OF)^R$	3	-	-	S81L	S79F

LEV: levofloxacin; NOR: norfloxacin; CIP: ciprofloxacin; PF: pefloxacin; OF: ofloxacin; $()^S$: susceptible; $()^R$: resistant; - : Variant without mutations. Phenotypic resistance profile is informed only of tested antibiotics.

Six out of the ten isolates were phenotypically susceptible to levofloxacin (LEV) and norfloxacin (NOR) but presented mutations in the QRDR of *gyr*A and/or *par*C associated with quinolone resistance in GBS (Hayes, O'Halloran, and Cotter 2020). The six isolates were recovered from urinary infections and presented MIC values for LEV of 0.5-1 µg/ml, as most of the LEV^S isolates with no mutations in *gyr*A or *par*C. Three out of the six GBS presented mutations in only one of the genes (first step mutation), which could explain the lack of phenotypic resistance. The other three isolates, however, presented second step mutations (mutations in both genes) typical of high level quinolone resistance, which is incongruent with the phenotypic results. It cannot be ruled out that these isolates are phenotypically resistant to other untested quinolones.

The remaining 4/10 isolates presented resistance to more than one quinolone (in all cases without inhibition halo in the disk diffusion test and with MIC values for LEV above 16 µg/ml) but no mutations were detected *in silico* in *gyr*A or *par*C. As second step mutations had been previously detected by PCR in 3/4 GBS, the QRDR of *gyr*A and *par*C were manually analyzed in the 4 genomes, but no point mutations were found. No explanation was found for this discrepancy.

Supplementary Figures

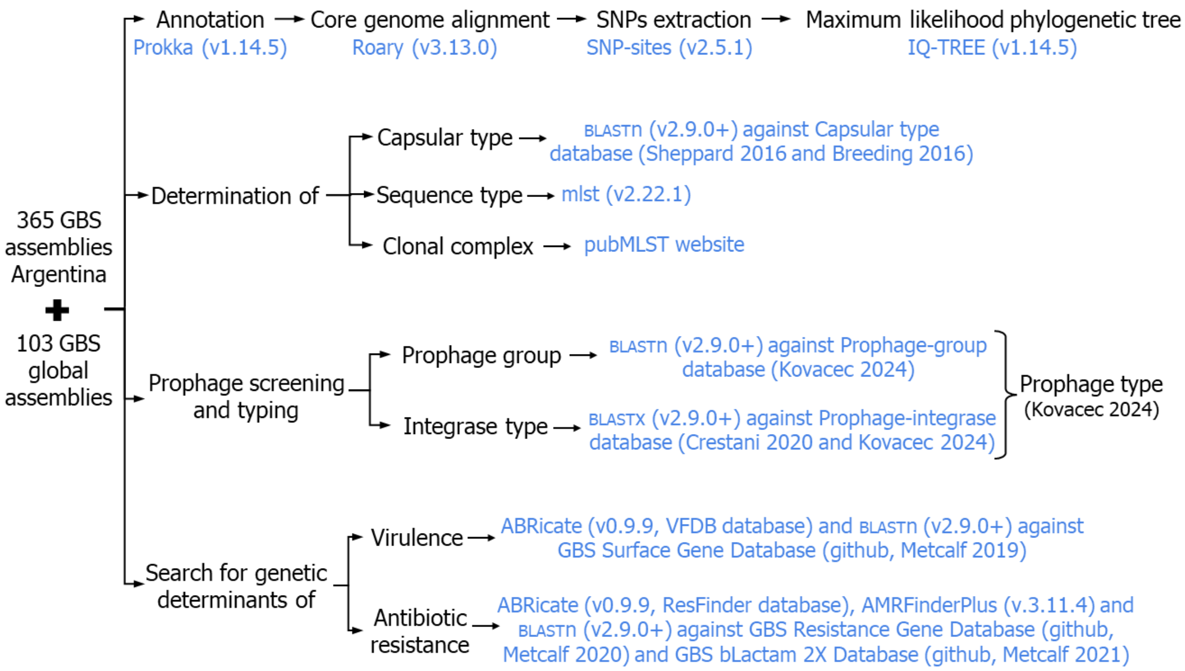

Supplementary Figure 1. Schematic of the genomic characterisation of the 468 GBS genomes of humans from 17 countries around the world. SNPs: single nucleotide polymorphisms.

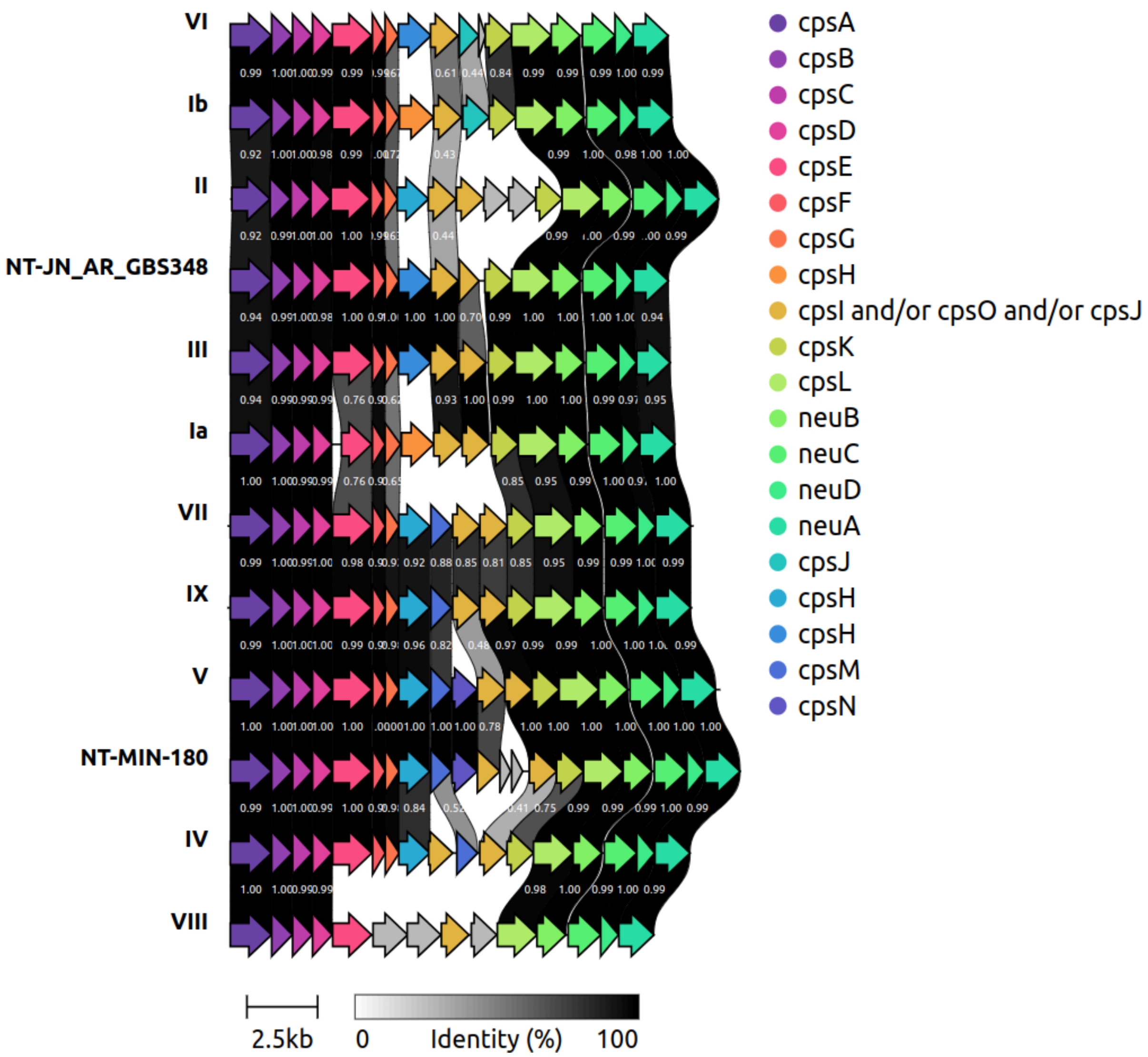

Supplementary Figure 2. Comparative analysis of the *cps* operon of representative sequences of each capsular type and two non-typeable strains. Colours represent groups of homologous genes. Genes with more than 40% of identity are linked with grey-black strokes, as shown in the scale. The homology percentage between genes is also shown inside each stroke.

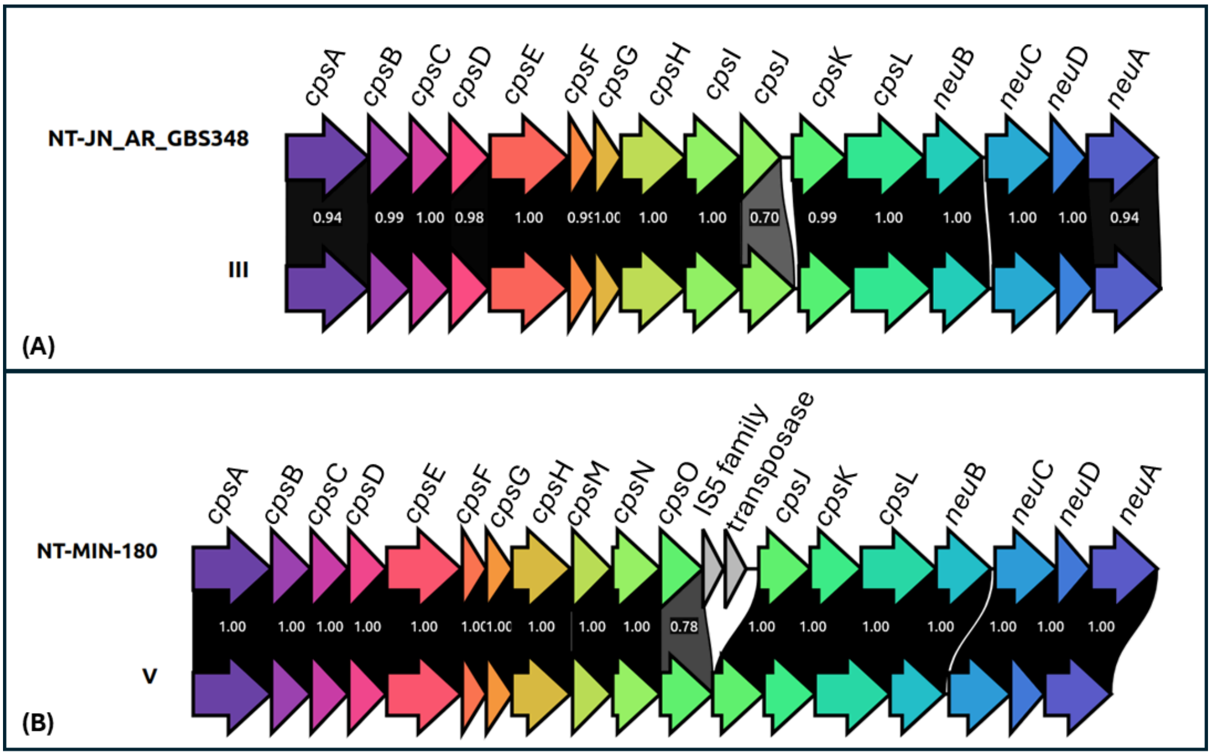

Supplementary Figure 3. Comparison of the *cps* operon of non-typeable (NT) strains and the closest related capsular type reference. (A) NT strain JN_AR_GBS348 vs reference strain of capsular type III. (B) NT strain MIN-180 vs reference strain of capsular type V. Colours represent groups of homologous genes. Genes with more than 40% of identity are linked with grey-black strokes and their homology is shown inside each stroke.

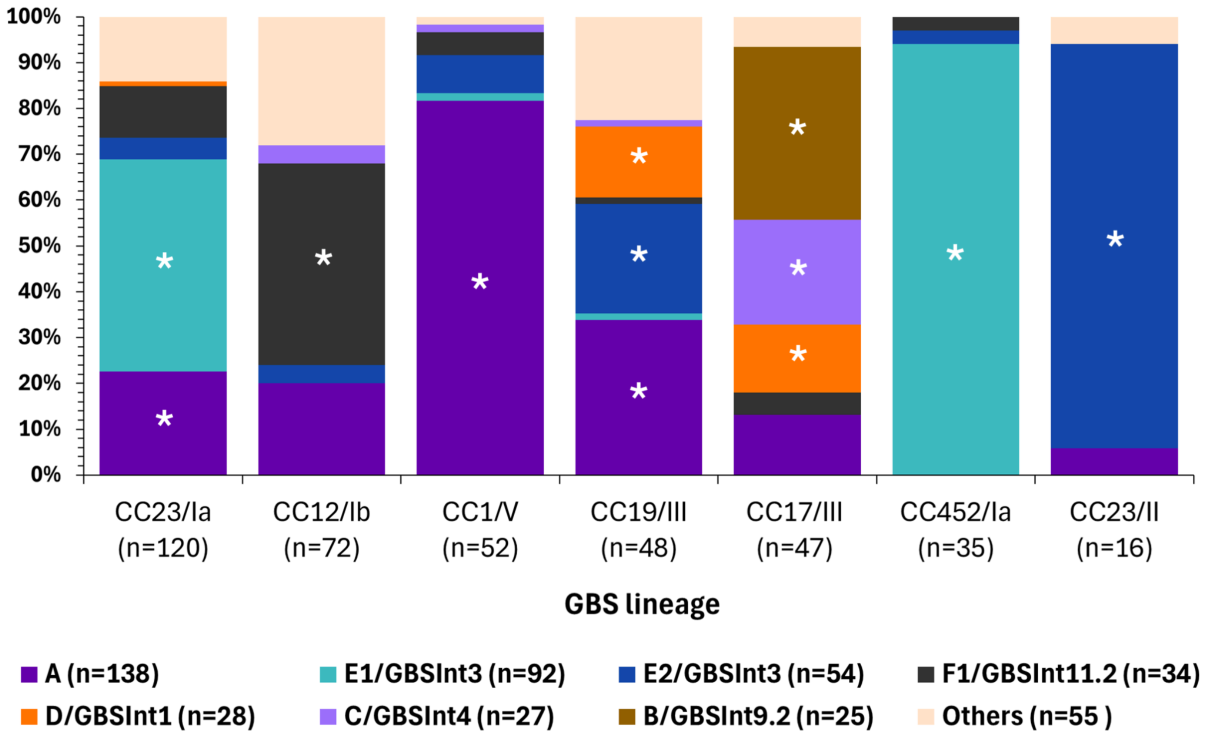

Supplementary Figure 4. Distribution of prophage types in the main lineages of GBS isolates. Lineages and prophage types present in more than 5% of the genomes are shown, in order of prevalence. Significant associations ($p<0.05$) between prophage type and lineage are marked with *.

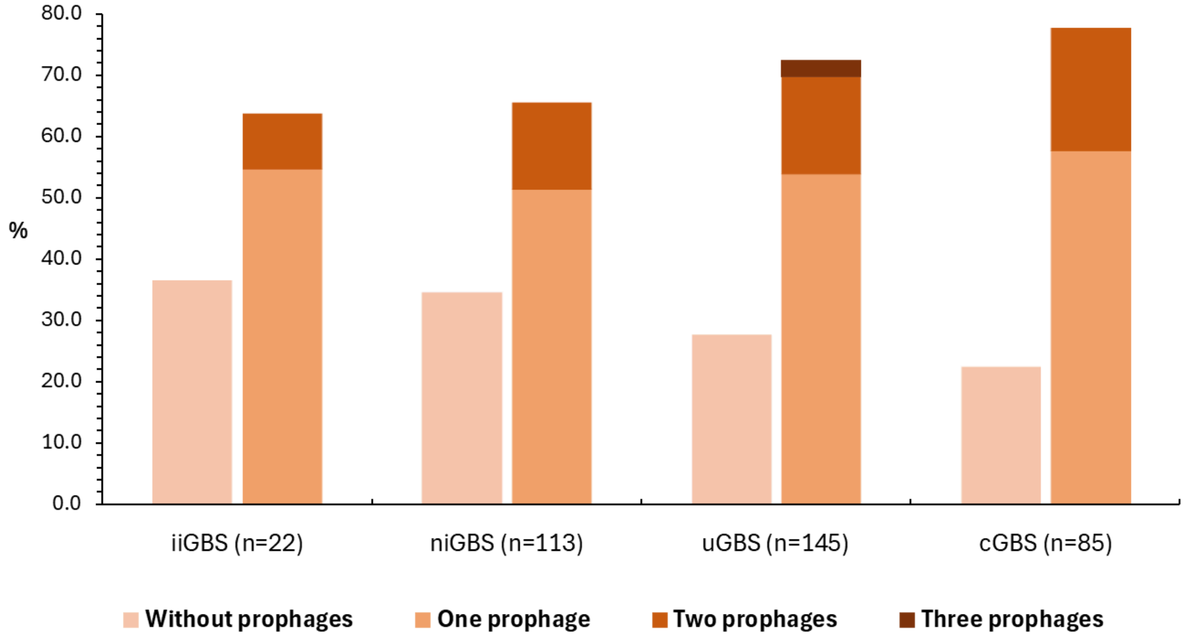

Supplementary Figure 5. Prophage presence per isolate in Argentinian GBS strains according to collection. iiGBS: GBS recovered from infant invasive infections; niGBS: GBS recovered from non-infant invasive infections; uGBS: GBS recovered from urinary infections; cGBS: GBS recovered from pregnant women during prenatal screenings.

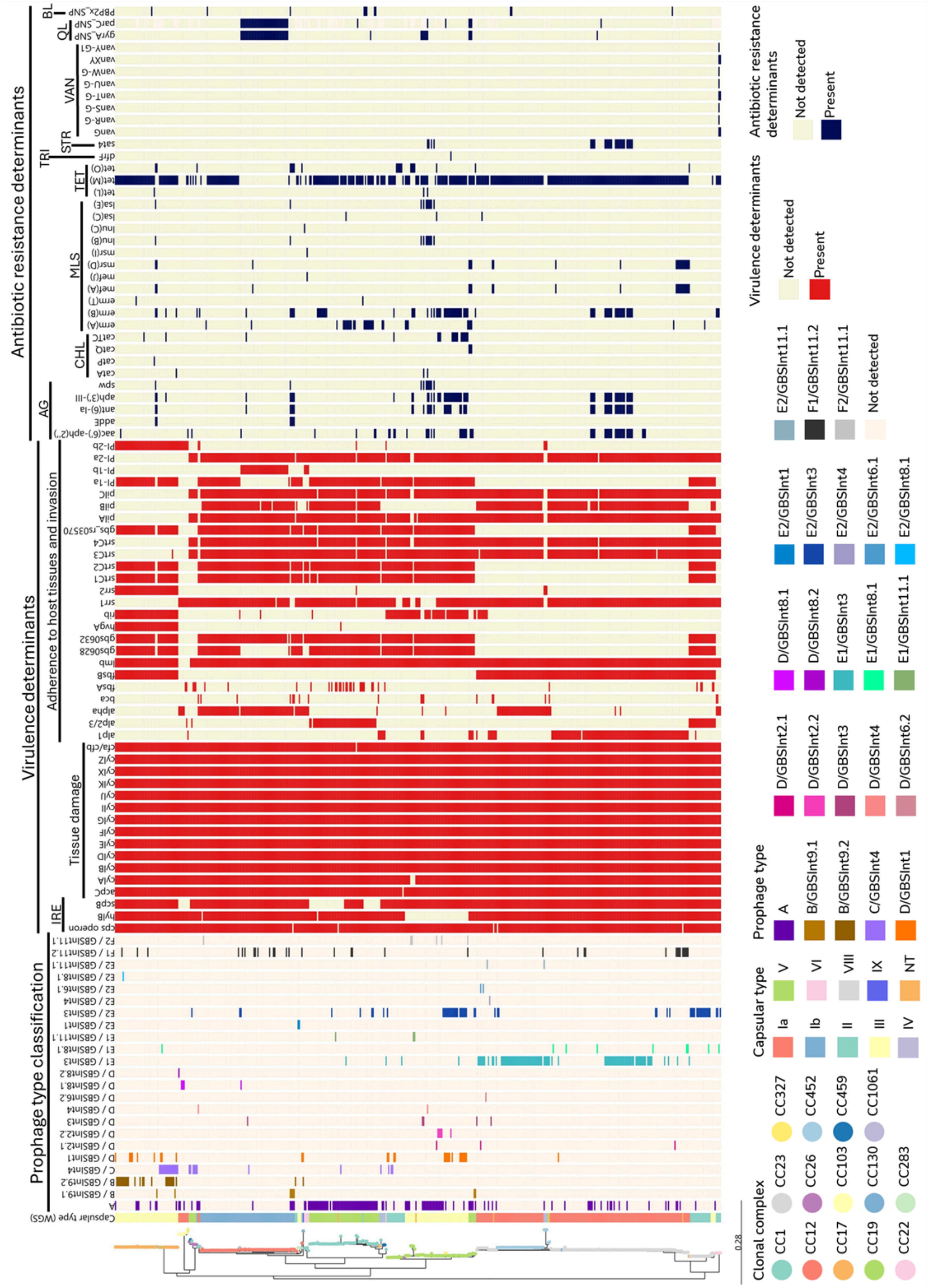

Supplementary Figure 6. Distribution in the phylogeny of prophages, virulence determinants and antimicrobial resistance determinants according to clonal complex and capsular type. Core-SNPs maximum likelihood phylogenetic tree, midpoint rooted, with nodes coloured by clonal complex. Strains metadata are shown as coloured blocks. IRE: Immune response evasion; AG: aminoglycosides; CHL: chloramphenicol; MLS: macrolides/lincosamides/streptogramins; TET: tetracyclines; TRI: trimethoprim; STR: streptothricin; VAN: vancomycin; QL: quinolones; BL: beta-lactams; NT: non-typeable. https://microreact.org/project/gbs-pangenomic-analysis

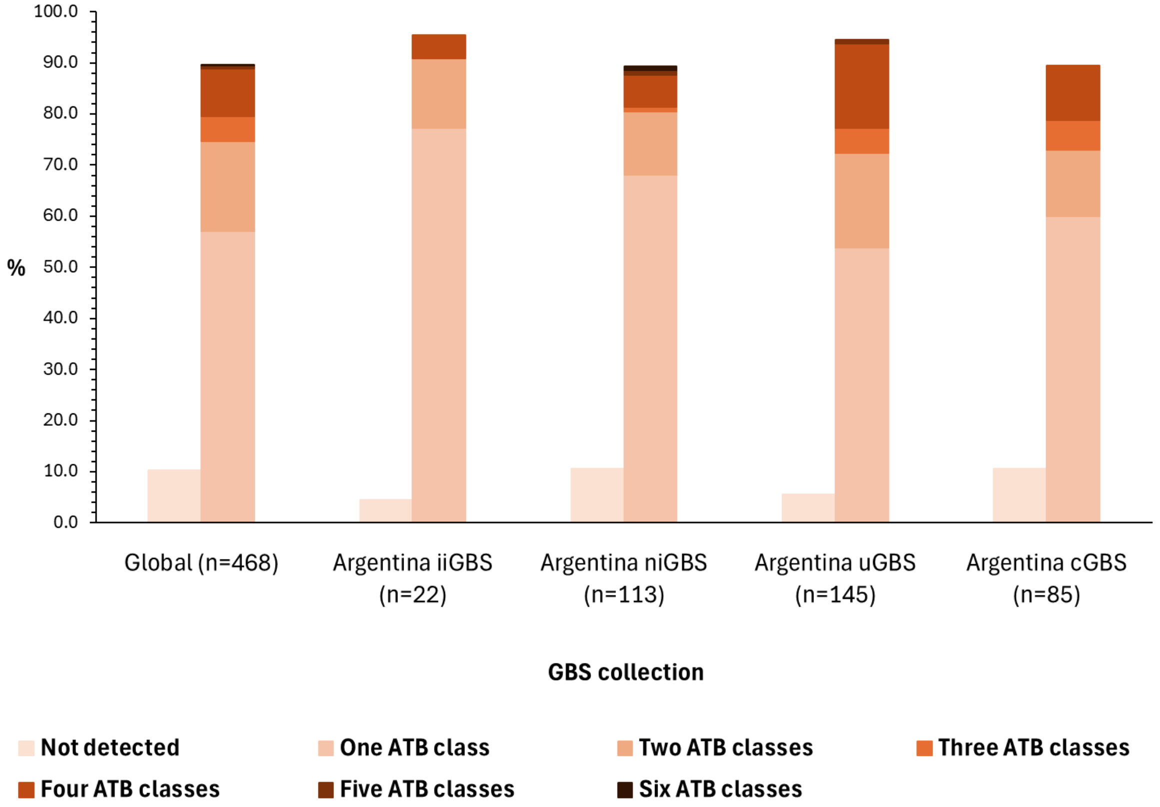

Supplementary Figure 7. Presence of resistance determinants to multiple antibiotic (ATB) classes in the 468 global GBS assemblies analysed and the Argentinian GBS discriminated by collection. iiGBS: GBS recovered from infant invasive infections; niGBS: GBS recovered from non-infant invasive infections; uGBS: GBS recovered from urinary infections; cGBS: GBS recovered from pregnant women during prenatal screenings.

Supplementary References

Al Safadi, Rim, Souheila Amor, Geneviève Hery-Arnaud, Barbara Spellerberg, Philippe Lanotte, Laurent Mereghetti, François Gannier, Roland Quentin, and Agnès Rosenau. 2010. “Enhanced Expression of Lmb Gene Encoding Laminin-Binding Protein in Streptococcus Agalactiae Strains Harboring IS1548 in scpB-Lmb Intergenic Region.” Edited by Frank R. DeLeo. *PLoS ONE* 5 (5): e10794. https://doi.org/10.1371/journal.pone.0010794.

Bobadilla, Fernando J., Marina G. Novosak, Iliana J. Cortese, Osvaldo D. Delgado, and Margarita E. Laczeski. 2021. “Prevalence, Serotypes and Virulence Genes of Streptococcus Agalactiae Isolated from Pregnant Women with 35–37 Weeks of Gestation.” *BMC Infectious Diseases* 21 (1): 73. https://doi.org/10.1186/s12879-020-05603-5.

Emaneini, Mohammad, Fereshteh Jabalameli, Akbar Mirsalehian, Amir Ghasemi, and Reza Beigverdi. 2016. “Characterization of Virulence Factors, Antimicrobial Resistance Pattern and Clonal Complexes of Group B Streptococci Isolated from Neonates.” *Microbial Pathogenesis* 99 (October):119–22. https://doi.org/10.1016/j.micpath.2016.08.016.

Eskandarian, N., Z. Ismail, V. Neela, A. Van Belkum, M. N. M. Desa, and S. Amin Nordin. 2015. “Antimicrobial Susceptibility Profiles, Serotype Distribution and Virulence Determinants among Invasive, Non-Invasive and Colonizing Streptococcus Agalactiae (Group B Streptococcus) from Malaysian Patients.” *European Journal of Clinical Microbiology & Infectious Diseases* 34 (3): 579–84. https://doi.org/10.1007/s10096-014-2265-x.

Granlund, Margareta, François Michel, and Mari Norgren. 2001. “Mutually Exclusive Distribution of IS *1548* and GBSi1, an Active Group II Intron Identified in Human Isolates of Group B Streptococci.” *Journal of Bacteriology* 183 (8): 2560–69. https://doi.org/10.1128/JB.183.8.2560-2569.2001.

Hayes, Katherine, Fiona O’Halloran, and Lesley Cotter. 2020. “A Review of Antibiotic Resistance in Group B Streptococcus: The Story so Far.” *Critical Reviews in Microbiology* 46 (3): 253–69. https://doi.org/10.1080/1040841X.2020.1758626.

Jamrozy, Dorota, Guduru Gopal Rao, Theresa Feltwell, Theresa Lamagni, Priya Khanna, Androulla Efstratiou, Julian Parkhill, and Stephen D. Bentley. 2023. “Population Genetics of Group B Streptococcus from Maternal Carriage in an Ethnically Diverse Community in London.” *Frontiers in Microbiology* 14 (May):1185753. https://doi.org/10.3389/fmicb.2023.1185753.

Lynskey, Nicola N., Mark Reglinski, Damien Calay, Matthew K. Siggins, Justin C. Mason, Marina Botto, and Shiranee Sriskandan. 2017. “Multi-Functional Mechanisms of Immune Evasion by the Streptococcal Complement Inhibitor C5a Peptidase.” Edited by Debra E Bessen. *PLOS Pathogens* 13 (8): e1006493. https://doi.org/10.1371/journal.ppat.1006493.

Madzivhandila, Mashudu. 2013. “Serotype, Pilus Island Distribution and Molecular Epidemiology of Streptococcus Agalactiae Isolates from Colonization and Invasive Disease.” Johannesburg: University of the Witwatersrand, Faculty of Health Sciences. chrome-extension://efaidnbmnnnibpcajpcglclefindmkaj/https://wiredspace.wits.ac.za/server/api/core/bitstreams/3bbf35ad-fa12-4af2-816e-c08eaf7fba8a/content.

Manning, Shannon D, Moran Ki, Carl F Marrs, Kiersten J Kugeler, Stephanie M Borchardt, Carol J Baker, and Betsy Foxman. 2006. “The Frequency of Genes Encoding Three Putative Group B Streptococcal Virulence Factors among Invasive and Colonizing Isolates.” *BMC Infectious Diseases* 6 (1): 116. https://doi.org/10.1186/1471-2334-6-116.

Martins, E. R., A. Andreu, J. Melo-Cristino, and M. Ramirez. 2013. “Distribution of Pilus Islands in Streptococcus Agalactiae That Cause Human Infections: Insights into Evolution and Implication for Vaccine Development.” *Clinical and Vaccine Immunology* 20 (2): 313–16. https://doi.org/10.1128/CVI.00529-12.

Pritzlaff, Craig A., Jennifer C. W. Chang, Shrin P. Kuo, Glen S. Tamura, Craig E. Rubens, and Victor Nizet. 2001. “Genetic Basis for the Β-haemolytic/Cytolytic Activity of Group B *Streptococcus*.” *Molecular Microbiology* 39 (2): 236–48. https://doi.org/10.1046/j.1365-2958.2001.02211.x.

Rolland, Karine, Corinne Marois, Veronique Siquier, Blandine Cattier, and Roland Quentin. 1999. “Genetic Features of *Streptococcus Agalactiae* Strains Causing Severe Neonatal Infections, as Revealed by Pulsed-Field Gel Electrophoresis and *hylB* Gene Analysis.” *Journal of Clinical Microbiology* 37 (6): 1892–98. https://doi.org/10.1128/JCM.37.6.1892-1898.1999.

Rosenau, Agnès, Karine Martins, Souheila Amor, François Gannier, Philippe Lanotte, Nathalie Van Der Mee-Marquet, Laurent Mereghetti, and Roland Quentin. 2007. “Evaluation of the Ability of *Streptococcus Agalactiae* Strains Isolated from Genital and Neonatal Specimens To Bind to Human Fibrinogen and Correlation with Characteristics of the *fbsA* and *fbsB* Genes.” *Infection and Immunity* 75 (3): 1310–17. https://doi.org/10.1128/IAI.00996-06.

Safadi, Rim Al, Laurent Mereghetti, Mazen Salloum, Marie-Frédérique Lartigue, Isabelle Virlogeux-Payant, Roland Quentin, and Agnès Rosenau. 2011. “Two-Component System RgfA/C Activates the fbsB Gene Encoding Major Fibrinogen-Binding Protein in Highly Virulent CC17 Clone Group B Streptococcus.” Edited by Adam J. Ratner. *PLoS ONE* 6 (2): e14658. https://doi.org/10.1371/journal.pone.0014658.

Schneewind, O, K Friedrich, and R Lütticken. 1988. “Cloning and Expression of the CAMP Factor of Group B Streptococci in Escherichia Coli.” *Infection and Immunity* 56 (8): 2174–79. https://doi.org/10.1128/iai.56.8.2174-2179.1988.

Seo, Ho Seong, George Minasov, Ravin Seepersaud, Kelly S. Doran, Ievgeniia Dubrovska, Ludmilla Shuvalova, Wayne F. Anderson, Tina M. Iverson, and Paul M. Sullam. 2013. “Characterization of Fibrinogen Binding by Glycoproteins Srr1 and Srr2 of Streptococcus Agalactiae.” *Journal of Biological Chemistry* 288 (50): 35982–96. https://doi.org/10.1074/jbc.M113.513358.

Tazi, Asmaa, Olivier Disson, Samuel Bellais, Abdelouhab Bouaboud, Nicolas Dmytruk, Shaynoor Dramsi, Michel-Yves Mistou, et al. 2010. “The Surface Protein HvgA Mediates Group B Streptococcus Hypervirulence and Meningeal Tropism in Neonates.” *Journal of Experimental Medicine* 207 (11): 2313–22. https://doi.org/10.1084/jem.20092594.

Tazi, Asmaa, Céline Plainvert, Olivia Anselem, Morgane Ballon, Valérie Marcou, Aurélien Seco, Fatma El Alaoui, et al. 2019. “Risk Factors for Infant Colonization by Hypervirulent CC17 Group B Streptococcus: Toward the Understanding of Late-Onset Disease.” *Clinical Infectious Diseases* 69 (10): 1740–48. https://doi.org/10.1093/cid/ciz033.

Tsai, I-An, Yaochi Su, Ying-Hsiang Wang, and Chishih Chu. 2022. “Alterations in Genes Rib, scpB and Pilus Island Decrease the Prevalence of Predominant Serotype V, Not III and VI, of Streptococcus Agalactiae from 2008 to 2012.” *Pathogens* 11 (10): 1145. https://doi.org/10.3390/pathogens11101145.

Udo, Edet E., Samar S. Boswihi, and Noura Al-Sweih. 2013. “Genotypes and Virulence Genes in Group B Streptococcus Isolated in the Maternity Hospital, Kuwait.” *Medical Principles and Practice* 22 (5): 453–57. https://doi.org/10.1159/000349932.

Wang, Zhaofei, Changming Guo, Yannan Xu, Guangjin Liu, Chengping Lu, and Yongjie Liu. 2014. “Two Novel Functions of Hyaluronidase from Streptococcus Agalactiae Are Enhanced Intracellular Survival and Inhibition of Proinflammatory Cytokine Expression.” Edited by S. M. Payne. *Infection and Immunity* 82 (6): 2615–25. https://doi.org/10.1128/IAI.00022-14.

Yildirim, A.Ö, K Fink, and Ch Lämmler. 2002. “Distribution of the Hyaluronate Lyase Encoding Gene hylB and the Insertion Element Is1548 in Streptococci of Serological Group B Isolated from Animals and Humans.” *Research in Veterinary Science* 73 (2): 131–35. https://doi.org/10.1016/S0034-5288(02)00029-2.
